# Choosing Clinical Variables for Risk Stratification Post-Acute Coronary Syndrome

**DOI:** 10.1038/s41598-019-50933-3

**Published:** 2019-10-10

**Authors:** Paul D. Myers, Wei Huang, Fred Anderson, Collin M. Stultz

**Affiliations:** 1 Department of Electrical Engineering and Computer Science and Research Laboratory for Electronics, Massachusetts Institute of Technology, Cambridge, MA USA; 2 Institute for Medical Engineering and Science, Massachusetts Institute of Technology, Cambridge, MA USA; 3 Center for Outcomes Research, University of Massachusetts Medical School, Worcester, MA USA; 4 Division of Cardiology, Massachusetts General Hospital, Boston, MA USA

**Keywords:** Cardiology, Medical research

## Abstract

Most risk stratification methods use expert opinion to identify a fixed number of clinical variables that have prognostic significance. In this study our goal was to develop improved metrics that utilize a variable number of input parameters. We first used Bootstrap Lasso Regression (BLR) – a Machine Learning method for selecting important variables – to identify a prognostic set of features that identify patients at high risk of death 6-months after presenting with an Acute Coronary Syndrome. Using data derived from the Global Registry of Acute Coronary Events (GRACE) we trained a logistic regression model using these features and evaluated its performance on a development set (N = 43,063) containing patients who have values for all features, and a separate dataset (N = 6,363) that contains patients who have missing feature values. The final model, Ridge Logistic Regression with Variable Inputs (RLRVI), uses imputation to estimate values for missing features. BLR identified 19 features, 8 of which appear in the GRACE score. RLRVI had modest, yet statistically significant, improvement over the standard GRACE score on both datasets. Moreover, for patients who are relatively low-risk (GRACE≤87), RLRVI had an AUC and Hazard Ratio of 0.754 and 6.27, respectively, vs. 0.688 and 2.46 for GRACE, (*p* < 0.007). RLRVI has improved discriminatory performance on patients who have values for the 8 GRACE features plus any subset of the 11 non-GRACE features. Our results demonstrate that BLR and data imputation can be used to obtain improved risk stratification metrics, particularly for patients who are classified as low risk using traditional methods.

## Introduction

Risk-stratification plays an important role in the management of patients after an acute coronary syndrome (ACS). Early identification of patients at risk of adverse outcomes helps to ensure that they are assigned therapies that are appropriate for their level of risk. For example, patients who are identified as high risk using traditional risk metrics benefit from invasive therapies within 24–48 hours of presentation^1,2^. Risk scores such as the Global Registry of Acute Coronary Events (GRACE) and the Thrombolysis in Myocardial Infarction (TIMI) scores are widely used metrics that are calculated using a patient’s presenting signs and symptoms, historical data – information that is available at the time of presentation – and the results of laboratory studies that can be obtained within minutes to hours after presentation^3–5^. Despite the relative success of these methods, an accurate assessment of patient risk remains a difficult task. Many risk scores, for example, fail to capture a significant number of deaths in certain patient cohorts^6,7^. Indeed, while patients who fall into the highest risk group have the highest prevalence of adverse outcomes, many adverse events occur in patients who are not classified as being high risk using conventional metrics. This happens because only a small fraction of the population is typically classified as being high risk by most risk scores. Although the probability of adverse outcomes in patients who are not-high risk is relatively low, the absolute number of deaths in this cohort will be large as the vast majority of the population is considered to be not high-risk. Such “low risk-high number” phenomena plague many risk stratification problems in medicine^6,8^. There is therefore a great need to develop risk models that can more accurately identify high risk subgroups that are missed by traditional metrics.

Traditional risk scores that are used in clinical practice were developed using regression models that take a fixed number of clinical variables as input. These input features are typically derived from an analysis of the relevant clinical literature and the opinions of experts who are well versed in the signs, symptoms, associated risk factors, and pathophysiology of ACS^3–5,9,10^. While “domain-specific knowledge” – information that can be garnered from clinical experts and pertinent scholarship – provides a powerful resource for identifying clinical variables that have prognostic value, domain experts are not infallible. As clinical information grows with time, for example, factors that were once deemed important may later be found to be less so, and factors that were once not thought to be significant may later be found to have prognostic value. We therefore hypothesized that feature selection methods that rely on limited domain specific information would identify important clinical characteristics that could be used to build risk metrics that are better able to identify high risk subgroups among patients who are identified as being low risk using traditional methods.

In this work we demonstrate that machine learning techniques can be used to select a subset of prognostic features from a large list of patient characteristics in an unbiased manner. We show that this naive approach can discover prognostic features that were not identified by domain experts when deriving the original GRACE score, thereby demonstrating the potential benefit of using methods that do not heavily rely on domain specific information. We also use a data imputation method to impute missing clinical variables, which enables the model to use a variable number of input parameters. We show that the resulting model can identify patients at risk of adverse events amongst cohorts that would not be classified as high risk using the original GRACE score.

## Methods

This study used the ACS cohort in the GRACE study and the outcome of interest was six-month all-cause mortality from admission. GRACE was designed to reflect an unbiased and generalizable sample of ACS patients hospitalized from 1999 to 2007 in 94 hospitals in 14 countries. All methods were carried out in accordance with relevant guidelines and regulations at each participating site, and only patients $\ge 18$ years of age were eligible to be enrolled in the database^11^. The GRACE protocol was approved by the UMass Medical School institutional review board and participating hospitals, where required, also received approval from their local ethics or institutional review boards. Signed, informed consent for follow-up contact was obtained from the patients at enrollment. For those sites using active surveillance for case identification, verbal or written consent was obtained from patients to review information contained in their medical charts. Details of the GRACE design, recruitment, and data collection are described elsewhere^5,11–14^.

### Feature selection with bootstrap lasso regression

We restricted our analysis to clinical features that are available within the first 24 hours after presentation, yielding 198 such features in the registry. These features collectively include laboratory data, patient demographic information, as well medications administered during the first hospital day. In GRACE there were 15,534 patients who had values for all 198 features. We used 80% (12,428) of these patients for the BLR analysis and left the remaining 20% (3,106) as a holdout set. Both sets of patients had the same mortality rate.

All features were normalized to fall between 0 and 1, inclusive, where 0 corresponds to the minimum value of that feature in the dataset and 1 corresponds to the maximum feature value. Features were taken directly from the registry, so no feature preprocessing was necessary. In Bootstrap Least Absolute Shrinkage and Selection Operator (LASSO) Regression (BLR), a logistic regression model is trained using repeated rounds of bootstrapping where some fraction of the data is used for training and the remaining fraction is used for testing^15^. Lasso regression models have the property that many of the feature weights in the model are forced to zero, leaving only the most important features in the final model (see Supplementary Information for details). As the features that are selected by lasso regression may differ depending on the precise dataset used for training, we only use features that are consistently retained (i.e., have non-zero weights) after many bootstrap iterations.

We trained a BLR model on the 198 features available within the first 24 hours using a bootstrapping procedure, where a random subset of 80% of the 12,428 patients was used for training and hyperparameter tuning; this process was repeated 100 times to generate 100 bootstrap splits. We ensured that each random 80% had the same percentage of patients who died as in the overall registry; i.e., each bootstrap was stratified with respect to death. Hyperpameter tuning was done using three-fold cross validation on the training sets. Features that had non-zero weights in at least 90% of the bootstrap splits were retained. Throughout this work we use the term “bootstrap split” to refer to a training-test set pair generated as described above.

To determine whether the final set of features chosen by BLR yielded a model that has similar discriminatory ability relative to the model trained with 198 features, we trained a logistic regression model using all 198 features and compared its performance to a model trained with only the features selected by BLR and tested them both on the holdout set of 3,106 patients. Both models were trained using L2-regularization (see Supplementary Methods for details) on the 12,428 patients used for the BLR analysis. To determine statistical significance, we randomly selected 20% of the patients in the holdout set and evaluated the performance of both models on this 20%; we repeated this process 10 times to generate confidence intervals.

### Model development and testing

Our final risk stratification model was developed using L2-regularized logistic regression (also known as Ridge Logistic Regression, RLR) using the features selected by BLR. RLR, unlike lasso regression, tends to assign non-zeros weights to all of the variables that are used as input. L2-regularization is a method that helps to prevent over-fitting the model to the training data (see Supplementary Methods for details). Hence, while BLR is used to select important features (it assigns zero weights to features that are not related to the outcome of interest), RLR is used to prevent overfitting once the important features have been chosen (it finds values for the weights that minimizes overfitting assuming all of the input features are important).

We performed 100 bootstrap rounds on a development set where for each round 80% of the patients were used for training and the remaining 20% was used for testing; i.e., 100% of the dataset is represented across each train/test split. As before, the training and testing sets were constrained to have the same percentage of deaths as in the overall dataset. The development set consisted of all patients in the ACS cohort of the GRACE dataset who had values for all of the features discovered by BLR.

We evaluated the model’s performance on bootstrapped test sets as well as several clinically important subgroups within each test set; i.e., patients with: 1) ST-elevation myocardial infarction (STEMI), 2) non-ST elevation MI (NSTEMI), 3) unstable angina (UA), and 4) a GRACE score ≤ 87 (the “low-risk” patients). We chose a cutoff of 87 to identify low risk patients because prior work suggests that this value captures patients who fall within the lowest tertile of risk for both NSTEMI and STE-ACS and yields an overall 6-month mortality less than 2%^12,13^. In our development set, patients who have a GRACE score ≤ 87 fall within the lowest 14% of risk.

Metrics used to evaluate the model performance include the area under the receiver operator characteristic curve (AUC or C-statistic), six-month hazard ratio (HR, highest vs. other quartiles), and two-category net reclassification index (NRI)^16^. AUCs, HRs and NRIs, are reported as the means across these 100 bootstrap trials. Upper and lower 95% confidence intervals for the HRs are reported as the means of the confidence intervals across bootstrap rounds.

### Data imputation

In instances where patients are missing values for some of the features used in the RLR model, a data imputation technique was applied to estimate them. Imputation was done using a multivariate normal distribution with mean and covariance estimated using the sample mean and covariance of the training set. Absent values in the test set were imputed by finding the corresponding feature values that maximize the conditional probability of the normal distribution given values for the features that are present. These values can be analytically computed once the mean and covariance matrix of the multivariate normal distribution are specified (see Supplementary Materials for details). We call the resulting model, which uses imputed values for missing model parameters, RLR with a Variable number of Inputs (RLRVI) because, from the standpoint of the user, the model can accommodate a variable number of input features.

We note that the RLRVI model reduces to the RLR model for patients who have values for all model parameters. Furthermore, we note that this imputation procedure makes no assumptions about the underlying pattern of “missingness” within the data. Rather it makes a fundamental assumption about the underlying distribution of the features; i.e., that they arise from a multivariate normal distribution.

### Testing on the validation set

To further evaluate the model, we constructed a validation set that comprised patients who had all eight GRACE score features, but who were not part of our development set nor part of the patient cohort that was used to derive the original GRACE score. Patients in the validation set could be missing any of the non-GRACE score features that are part of the RLRVI model. We trained a RLRVI model on the entire development set, and evaluated its performance on the validation set. Thus, the validation set was used as a held-out test set; no feature selection or model development was done on this group of patients. For comparison, we also evaluated the performance of the GRACE score on the validation set. To obtain confidence intervals for both models, we computed performance metrics (e.g., AUCs, HRs) using bootstrapping. For each bootstrap iteration, we randomly chose 20% of the validation set (subject to the constraint that it had the same percentage of deaths as in the overall validation set) and evaluated the models’ performance on this subset. The confidence intervals were calculated as the standard error of the mean (standard deviation divided by the square root of the number of bootstrap splits) for the AUCs and HRs across these 100 bootstrap splits.

### Statistical analyses

HRs were computed using a Cox proportional hazards model^17^. Confidence intervals were generated by computing the standard error of the mean from the bootstrap test sets. HRs were computed by placing all patients with model scores in the upper-quartile of risk in the high-risk group and all other patients in the not-high-risk group. The cutoff for determining the upper-quartile score was derived from the training sets. Statistical significance testing was done using two-sided, paired-sample *t*-tests between each pair of models over the 100 bootstrap splits. All logistic regression models and statistical analyses were performed using the commercial software MATLAB 9.0 (2016a) (The MathWorks, Natick, MA).

## Results

### Feature selection with BLR

BLR identified 19 clinical features out of 198 clinical features available within the first 24 hours as being the most predictive (Table 1). A L2 regularized model using the 19 features has an AUC on the holdout set of 0.852, which is similar to the AUC of a L2 regularized model using all 198 features (AUC 0.859, p = 0.127).

**Table 1:** Features selected by the bootstrap lasso.

Demographics	Appears in GRACE Score?
Age	Yes
Admission weight	No
**Medical History**	
Congestive heart failure	No
Peripheral artery disease	No
Renal insufficiency	No
**Presentation Characteristics**	
Systolic blood pressure	Yes
Pulse	Yes
Killip class	Yes
Cardiac arrest	Yes
ST segment deviation	Yes
**Medications, Chronic**	
Warfarin	No
Medications, pre-hospital or within 1^st^ 24 hours	
Statin	No
Diuretic	No
Insulin	No
IV inotropic agent	No
Oral beta blocker	No
IV beta blocker	No
**Laboratory**	
Initial creatinine	Yes
Initial positive enzymes	Yes

GRACE = Global Registry of Acute Coronary Events; IV = intravenous.

### RLRVI performance on the development set

A development set of 43,063 patients was constructed by collecting all patients who had values for all features identified by BLR. Table 2 shows the distribution of patient characteristics in the development set.

**Table 2:** Population characteristics in the development and validation sets.

	Development Set	Validation Set
Population size	43,063	6,363
Low-risk (GRACE score ≤ 87)	13,205	1,665
Mortalities	3,078 (7.15%)	719 (11.3%)
Low-risk (GRACE score ≤ 87)	316 (1.16%)	29 (1.74%)
**Demographics**		
Age (years)	66.1 (55.7–75.8)	68.2 (57.1–77.6)
Female	32.6%	33.9%
Height (cm)	170 (162–175)	169 (161–175)
Admission weight (kg)	77.0 (67.0–88.0)	77.0 (67.2–87.2)
Medical History (%)		
Congestive heart failure	10.5	11.1
Peripheral artery disease	9.7	9.2
Angina	51.9	45.5
Coronary Artery Bypass Graft (CABG)	12.6	11.9
Myocardial Infarction (MI)	30.3	31.0
Hypertension	62.1	61.6
Hyperlipidemia	48.3	48.1
Diabetes	25.1	26.3
Percutaneous Coronary Intervention (PCI)	17.7	17.7
Smoking	57.7	53.0
TIA/Stroke	8.3	9.1
Renal insufficiency	7.8	8.0
**Presentation Characteristics**		
Systolic blood pressure (mmHg)	140 (120–160)	140 (120–160)
Pulse (bpm)	77 (65–90)	77 (65–90)
Killip class I	83.3%	81.6%
Killip class II	12.0%	12.6%
Killip class III	3.9%	4.6%
Killip class IV	0.8%	1.3%
Cardiac arrest	1.7%	2.3%
ST segment deviation	54.8%	53.1%
**Medications (%)**		
Oral beta blocker, pre-hospital acute or within 1^st^ 24 hours in hospital	69.8	67.1
Warfarin, chronic use	4.5	5.2
Statin, pre-hospital acute or within 1^st^ 24 hours in hospital	51.0	56.6
Diuretic, pre-hospital acute or within 1^st^ 24 hours in hospital	25.3	28.0
Insulin, pre-hospital acute or within 1^st^ 24 hours in hospital	14.3	16.0
IV inotropic agent, pre-hospital acute or within 1^st^ 24 hours in hospital	4.5	6.3
IV beta blocker, pre-hospital acute or within 1^st^ 24 hours in hospital	12.9	11.5
Aspirin, within 1^st^ 24 hours in hospital	90.3	86.6
ACE Inhibitors, pre-hospital acute or within 1^st^ 24 hours in hospital	47.6	47.4
**Laboratory**		
Initial creatinine (mg/dl)	1.0 (0.9–1.3)	1.0 (0.9–1.3)
Initial positive enzymes	46.8%	50.7%

Numbers for continuous variables are presented as the median with the interquartile range in parentheses. GRACE = Global Registry of Acute Coronary Events; CABG = coronary artery bypass grafting; MI = myocardial infarction; PCI = percutaneous coronary intervention; TIA = transient ischemic attack; IV = intravenous; ACE = angiotensin converting enzyme.

**Figure 1:**
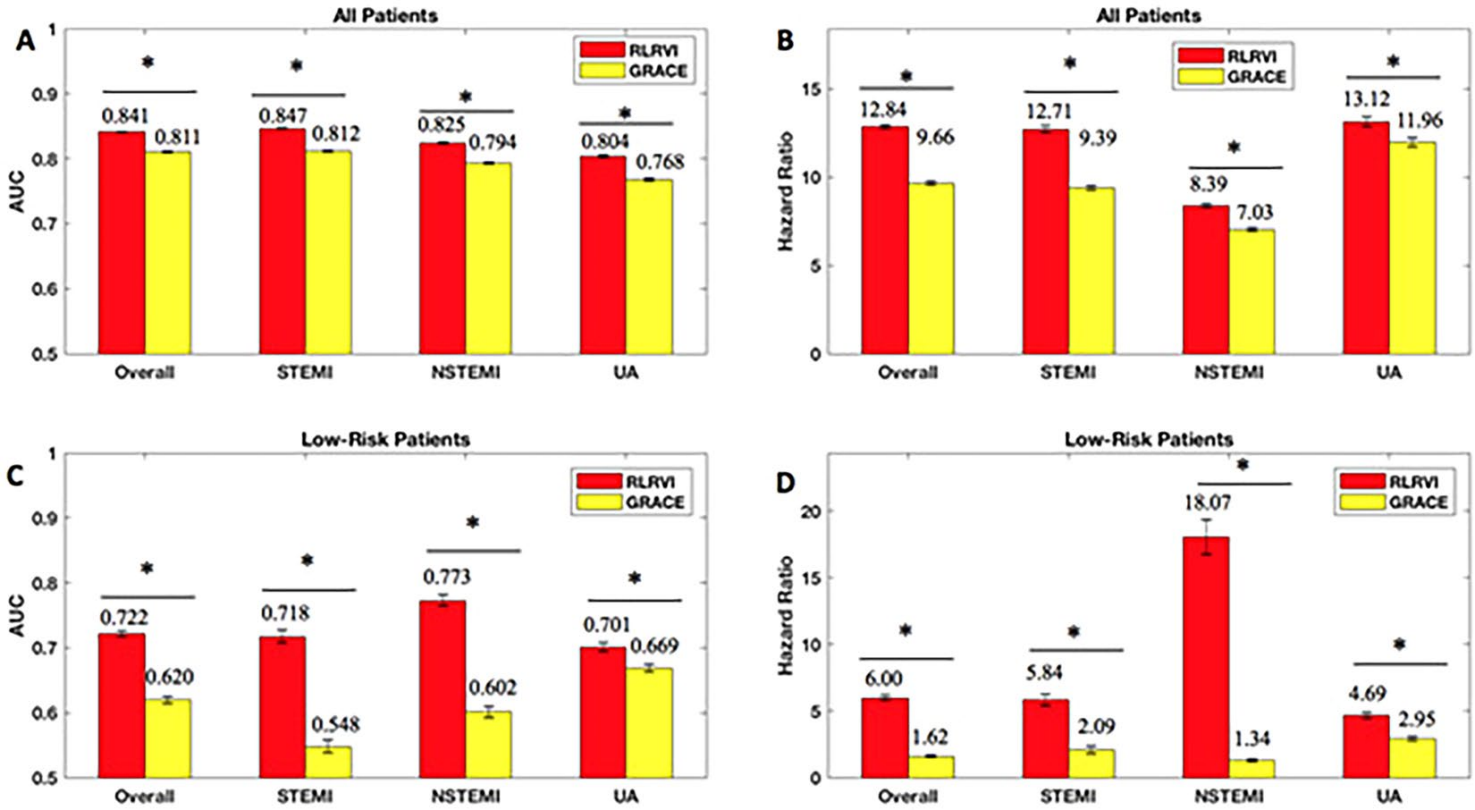
RLR Performance on the Development Set. AUCs and six-month hazard ratios in the overall (**a**,**b**) and low-risk (GRACE < 87) subset (**c**,**d**) of the development set. Error bars show one standard error of the mean. * indicates *p* < 0.001. Numbers above the bars indicate mean values. AUC = area under the curve; GRACE = Global Registry of Acute Coronary Events; RLRVI = ridge logistic regression with variable inputs; STEMI = ST elevation myocardial infarction; NSTEMI = non-ST elevation myocardial infarction; UA = unstable angina.

The RLRVI model has improved discriminatory ability relative to the GRACE score in the bootstrapped test sets, as well as the STEMI, NSTEMI, and UA subsets, as measured by the AUC (Fig. 1A). Similarly, HRs for all subsets show a statistically significant improvement over the GRACE score (Fig. 1B). The RLRVI model correctly classifies more patients than GRACE in all patient subsets, as evidenced by a positive two-category NRI of 0.0337 (standard deviation over 100 bootstrapped test sets 0.0149). Most notably, in patients who fall within the lowest 14% of risk (GRACE score ≤ 87), the RLRVI model yields significant improvements in both the discriminatory performance (Fig. 1C) and HR (Fig. 1D) relative to the GRACE score.

### Evaluating the relative importance of non-GRACE score features

**Figure 2:**
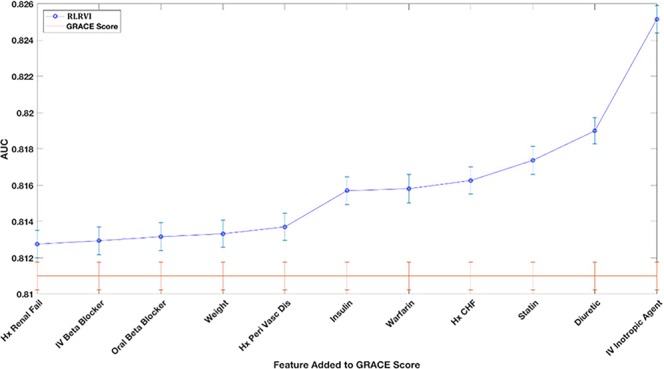
Evaluating the Relative Importance of non-GRACE Score Features. AUCs from adding one of the 11 non-GRACE score features at a time and imputing the remaining features. AUCs are averaged over 100 bootstrapped test sets. Error bars show one standard error of the mean. All models show improved performance over the GRACE score with *p* < 0.001. AUC = area under the curve; GRACE = Global Registry of Acute Coronary Events; RLRVI = ridge logistic regression with variable inputs; Hx = History; Peri Vasc Dis = peripheral vascular disease; IV = intravenous; CHF = congestive heart failure.

To determine the relative importance, over the standard GRACE score features, of each of the non-GRACE features in our model, we computed performance metrics for the RLRVI model when only the 8 GRACE score features plus one non-GRACE score feature was used as input. As the RLRVI model can accommodate 19 features as input, values for the remaining 10 features were estimated using the data imputation approach described in the methods section and in the Supplementary Materials. Adding each of the 11 non-GRACE score features to the 8 GRACE score features yield AUCs that are improved relative to the GRACE score values (Fig. 2).

### RLRVI discriminatory ability using a subset of clinical features

We sought to determine how the RLRVI model performs when the number of available clinical variables changes. If there are N < 19 features available, we could compute AUCs for the RLRVI model when only those N features are available, using data imputation to estimate the missing parameters. To determine what subsets of clinical features (if any) yield improvement when added to the established 8 GRACE score features, we considered all possible combinations of the remaining 11 features, giving 2,047 possible models.

**Figure 3:**
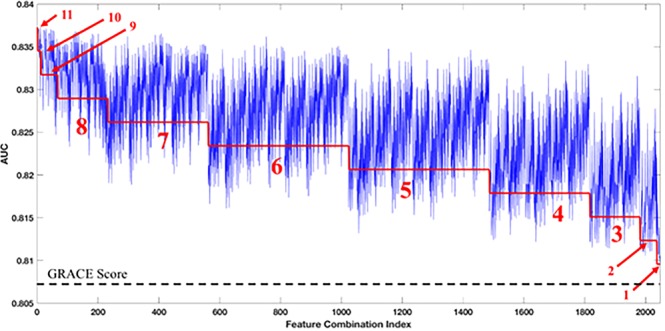
RLRVI Discriminatory Ability Using a Subset of Clinical Features. AUCs averaged over 10 bootstrap splits of the development set for all possible combinations of the 11 non-GRACE score features selected by BLR. The red line and numbers indicate the number of features that were known and therefore not imputed. For example, the red 6 indicates that all points in that range were generated by models that had six of the non-GRACE score features available; all possible combinations of 11 choose 6 are represented in this range. The performance of the GRACE score on the same 10 bootstrap splits is shown by the dashed line at the bottom of the plot. All feature combinations show improvement over the GRACE score with *p* < 0.003. AUC = area under the curve; GRACE = Global Registry of Acute Coronary Events.

We trained and tested each feature combination over 10 bootstrap splits of the development set. Figure 3 shows the AUC averaged over the 10 bootstrap splits for each possible feature combination. For all models, the RLRVI model provides improved discriminatory ability relative to the GRACE score with *p* < 0.003.

### RLRVI performance on the validation set

A validation set, which contains patients who were not used to develop either the RLRVI model or the original GRACE risk model, was used to further assess the model’s performance. Table 2 shows the distribution of patient characteristics in the development and validation sets.

**Figure 4:**
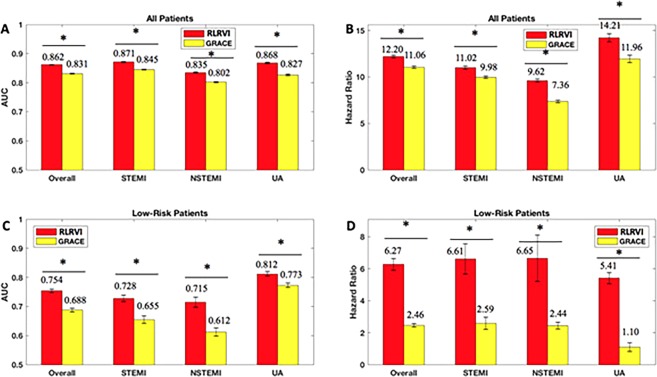
RLRVI Performance on the Validation Set. AUCs and six-month hazard ratios in the overall (**a**,**b**) and low-risk (GRACE < 87) subset (**c**,**d**) of the validation set. Error bars show one standard error of the mean. * indicates *p* < 0.007. Numbers above the bars indicate mean values. AUC = area under the curve; GRACE = Global Registry of Acute Coronary Events; RLRVI = ridge logistic regression with variable inputs; STEMI = ST elevation myocardial infarction; NSTEMI = non-ST elevation myocardial infarction; UA = unstable angina.

The RLRVI model has improved discriminatory ability relative to the GRACE score (Fig. 4A) and offers statistically significant improvement in all HRs in the validation set except the UA subgroup (Fig. 4B). The two-category NRI on the validation set is 0.0191, however, the standard deviation over 100 bootstrapped test sets is high (0.0315). In the lowest risk patients, the RLRVI model also has improved discriminatory ability in all patient subgroups (Fig. 4C) and offers improved HRs in all but the UA subgroup (Fig. 4D). Parameters for the final model are listed in the Supplementary Materials.

## Discussion

Risk stratification models that are used in clinical practice are often constructed using regression models that take a fixed number of clinical variables as input^3–5,9,10^. Variables that are thought to have prognostic significance are identified using a combination of expert opinion to first identify potential clinical characteristics followed by stepwise regression^18^. For example, the GRACE dataset – a registry derived from 94 hospitals across the globe – contains over 1400 clinical variables. In order to develop a risk model that could be used in clinical practice, a small subset of clinical variables (usually less than 50 features), which are typically available at presentation, was chosen based on published results from prior studies and expert clinical opinion^5,12,13^. Features in this list that had the greatest association with all-cause mortality were then selected and backward elimination was used to arrive at a regression model that included 14 features. A simpler model, which only uses 8 features that contain the most predictive information, was then provided for clinical use^13^.

As backward elimination, and, more generally stepwise regression, involves evaluating the performance of many models that contain different numbers of explanatory variables, the process becomes intractable when a large number of candidate variables are considered. For example, backward elimination using the 198 candidate variables we considered in this work would require training over 19,000 models to explore the different possible subsets of clinical variables. In these situations, expert knowledge forms an effective platform for limiting the number of potential variables, thereby a making comprehensive stepwise regression computationally tractable. While expert knowledge is a powerful resource that can be leveraged to identify the most important prognostic features, relying too heavily on expert opinion limits our ability to discover previously unappreciated characteristics that have prognostic value. We therefore implemented a feature selection technique based on a machine learning method called BLR^15^. Unlike traditional stepwise elimination, BLR can accommodate large feature sets, thereby eliminating the need to use expert knowledge to pre-prune the feature set before feature selection. In the present study, BLR identified 19 prognostic features from our original list of 198 by constructing only 100 models from 100 bootstrap rounds; i.e., one model for each bootstrap iteration. The fact that eight of the 19 features identified by BLR also appear in the GRACE score further supports the validity of using this automated feature selection method to generate feature sets (Table 2). Moreover, one of these 19 features (chronic warfarin use), although available at patient presentation, was not considered in the full GRACE model that included ~48 features that were identified by clinical experts, thereby demonstrating the ability of the method to discover new features with prognostic significance.

Although BLR, in general, facilitates the identification of a small set of features that can be used to build parsimonious risk stratification models, its effectiveness is limited in cases where features are highly correlated. For instance, given two important collinear features, BLR might select one in 50% of the bootstrap splits and the other in the remaining 50% of the splits. Moreover, as the method focusses on features that are consistently selected across different bootstrap splits, neither of these features would be identified as being important. While comprehensive stepwise regression avoids this problem by explicitly building models that include all possible feature combinations, it is associated with a significant computational cost when the number of potential features is large. Therefore, while BLR is not guaranteed to find all important clinical features, it does identify a subset of features that can be used to build effective risk stratification models. In this work, the model developed using only 19 features – representing less than 10% of the features available within 24 hours of presentation – has similar performance relative to the model developed using all 198 features. Models that utilize a parsimonious list of features are often easier to interpret and less likely to be over fit to a given training set.

The RLRVI model has improved discriminatory ability relative to the GRACE score and is better able to identify high risk patients as evidenced by the fact that high risk patients in the RLRVI model have higher Hazard Ratios relative to high risk patients in the GRACE model. While the overall improvement in discriminatory ability is modest, the greatest improvement occurs in patients that are at the lowest risk as defined by the original GRACE score, thereby demonstrating the prognostic power of the feature set on patients who are traditionally difficult to risk stratify. Indeed, the challenge of identifying high risk subgroups in patient cohorts that are traditionally thought of as being low risk is highlighted by our data. In the development set, 30% of patients fall into the low risk group and the corresponding death rate is 1.16% - significantly below the death rate in the entire dataset. However, failing to identify high risk patients in this low risk cohort would miss 10% of the total number of deaths. In this population the RLRVI model has improved discriminatory ability, and is better able to identify high risk patients as the HRs for the RLRVI are significantly larger than that of the GRACE score. This trend holds true in patients who present with STEMI or NSTEMI in both the development and validation datasets.

Although RLRVI uses 19 clinical features, data imputation allows the model to still yield predictive information when only subset of the 19 clinical features are available. Traditional risk scores, by contrast, can only be used when all of the input variables are known. To make such traditional models broadly applicable, they must therefore rely on features that are universally available at the time of admission. Moreover, an added constraint for models developed before the wide spread adoption of electronic health information systems was that the input variables needed to be manually entered by the relevant health care provider. Risk models like the original GRACE score, were therefore constructed to balance both predictive power and ease of manual use. Our model, which accommodates a variable number of input parameters, allows one to supply as much information as available to maximize its predictive ability. Central to the success of our model is a data imputation approach that allows us to estimate values for clinical variables when any of the 19 model parameters are not input. Interestingly, our data suggests that high imputation accuracy is not needed to obtain improvement in the model’s predictive ability over the GRACE score (Supplementary Materials Fig. S1 and Fig. 2). Moreover, while adding any subset of the non-GRACE score features to the 8 GRACE features yields a model with improved discriminatory ability, to achieve an optimal result the features with the most prognostic value should be included, in accordance with the trend shown in Fig. 2. Given the advent of electronic health records, models that utilize a large number of features become realizable. For such approaches to be maximally useful, they must be applicable to patients who have missing values for some input parameters, and our approach provides an example of how this can be achieved using a simple imputation method.

Risk stratification remains a challenging problem in patients with cardiovascular disease. This work highlights one approach for finding subsets of patient features that have prognostic value. We demonstrate that BLR can identify a small subset of prognostic features from among a much larger set of possibilities. The risk model arising from these features has improved performance relative to the original GRACE score, most notably for patients who are classified as being low risk using the original GRACE model. Our model can be used with the 8 GRACE score features plus any subset of the non-GRACE score variables, thereby enabling risk assessment when only a subset of the model’s features is known. It is our view that this work presents a new platform for the development of powerful risk stratification metrics.

## Supplementary information

Supplementary Materials
