# Supplementary material for: Choosing Clinical Variables for Risk Stratification Post-Acute Coronary Syndrome
Source: Sci Rep. 2019 Oct 10;9:14631. doi: 10.1038/s41598-019-50933-3 (PMC6787006; doi:10.1038/s41598-019-50933-3)
Supplement: Supplementary file 1 — Supplementary Materials [file 41598_2019_50933_MOESM1_ESM.docx]

**Supplementary Materials for**:

Choosing Clinical Variables for Risk Stratification Post-Acute Coronary Syndrome

**Brief title:** Choosing Clinical Variables with Machine Learning

Paul D. Myers B.S.E.^a^, Wei Huang, Ph.D. ^b,c^, Frederick A. Anderson Jr. Ph.D. ^b,c^, Collin M. Stultz M.D., Ph.D.^a,b,d^

*Bootstrap Lasso Regression*

In Bootstrap Least Absolute Shrinkage and Selection Operator (LASSO) Regression (BLR), a logistic regression model is trained using repeated rounds of bootstrapping where some fraction of the data is used for training and the remaining fraction is used for testing (14). Parameters for the model are obtained using the following relation:

$$\hat{w}=\mathop{\mathrm{argmin}}\limits_{(w_{1},...,w_{M})} \left\{\frac{1}{N}\sum_{i=1}^{N} \left[y_{i}\log\left(\hat{y}_{i}\right)+(1-y_{i})\log\left(1-\hat{y}_{i}\right)\right]+\lambda\sum_{j=1}^{M} \left|w_{j}\right|\right\}$$

where *N* is the number of patients, $(w_{1},...,w_{M})$are the $M$ model weights (one weight for each feature in the model) and $\hat{w}=(\hat{w}_{1},...,\hat{w}_{M})$ is the corresponding “optimal” value that solves the model; $y_{i}$ is the true label for patient $i$ ($y_{i}$ = 1 if patient $i$ died in 6 months and 0 otherwise), $\hat{y_{i}}$ is the output of the logistic regression model for patient $i$ and is therefore a function of the $M$ model weights, and $\lambda$ is a regularization parameter. L2-regularized logistic regression (also known as Ridge Logistic Regression, RLR) differs from Lasso regression in the form of the function to be optimized. Parameters for L2-regularized logistic regression are obtained as follows:

$$\hat{w}=\mathop{\mathrm{argmin}}\limits_{(w_{1},...,w_{M})} \left\{\frac{1}{N}\sum_{i=1}^{N} \left[y_{i}\log\left(\hat{y}_{i}\right)+(1-y_{i})\log\left(1-\hat{y}_{i}\right)\right]+\lambda\sum_{j=1}^{M} w_{j}^{2}\right\}$$

where each of the variables are defined as described above for Eq. . Eq. and Eq. differ only in the regularization term. L2-regularization, unlike lasso regression, tends to assign non-zeros weights to all of the variables that are used as input. For both Lasso and L2 methods, The regularization parameter, $\lambda$, was obtained using three-fold cross-validation on each training split.

*Data Imputation*

Our data imputation algorithm models the data as arising from a multi-variable normal distribution. Missing values are imputed by finding the values for the missing clinical variables that maximize the probability of the normal distribution given the values for the known features.

To evaluate the ability of out imputation method, we could delete all possible subsets of features for a given patient in the development set and impute values for the deleted features. However, for a given patient this would require $\sum_{k=1}^{19} \left(\begin{matrix} 19 \\ k \end{matrix}\right)=\text{524,287}$ imputation experiments. Repeating this process for all patients in the development set would require more than 20,000,000,000 imputation experiments.

To make the calculations more tractable, we randomly select subsets of features in the development set and delete the corresponding values. We then use our imputation procedure to estimate the values for the missing clinical variables. The procedure is repeated for different amounts of missing data; i.e., when 10%, 20%, 30%, …, 90% of the data are deleted. The results of all these imputation experiments are used to determine the average error/accuracy for imputing each of the 19 clinical variables given some number of known (i.e., not deleted) clinical features. The results for each of the 19 clinical variables are shown below (Fig S1A-S). Binary variables that can be imputed with high accuracy (>95%) include the presence of renal insufficiency, history of cardiac arrest, chronic warfarin use, and IV inotropic use. Most other binary variables are imputed with an accuracy >85%. Binary features with low imputation accuracy (50-75% accuracy) include a history of statin use, positive initial enzymes, ST segment changes, and oral beta-blocker use. Our data suggests that high accuracy for the imputing all model parameters is not needed to obtain improvement in the model’s predictive ability over the GRACE score (see Fig. 2 in main text).

Continuous Variables:


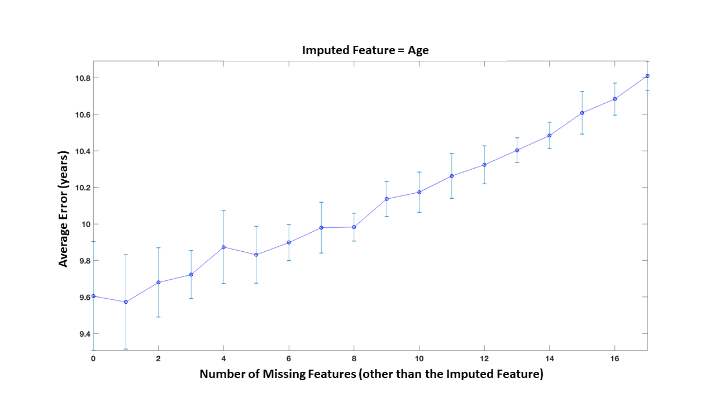

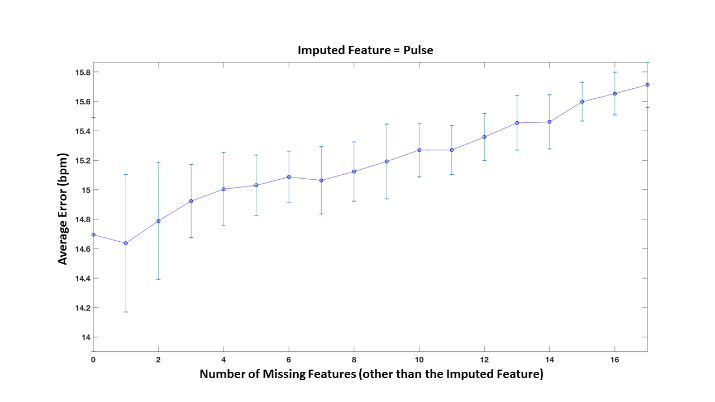

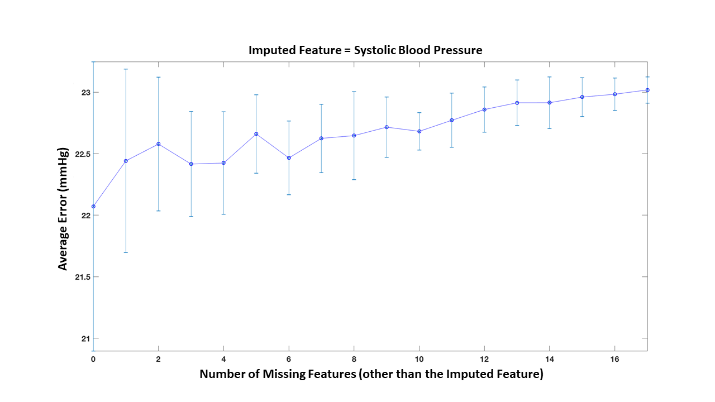

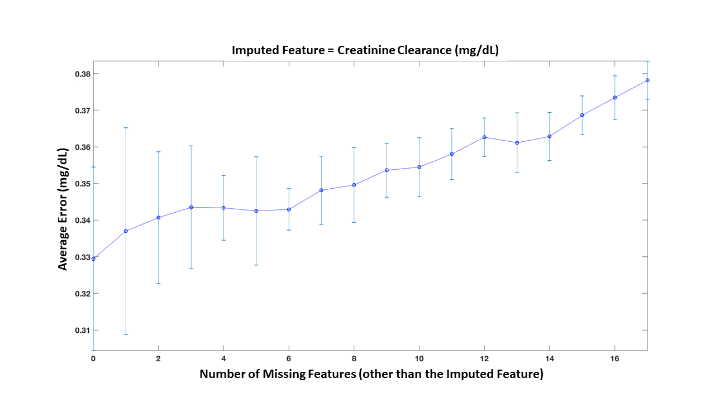


**A**

**B**

**C**

**D**


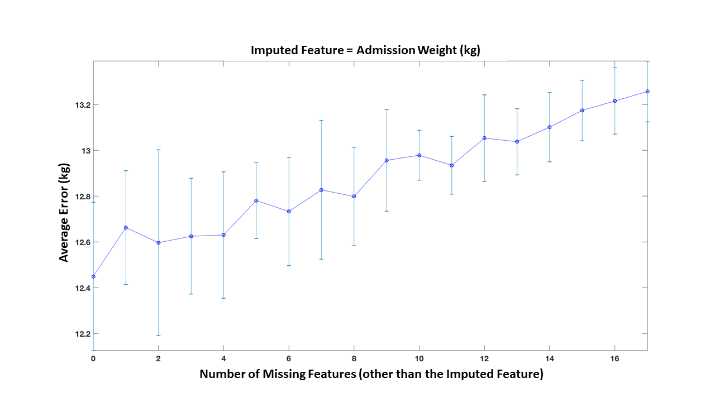


**E**

Categorical Variable:


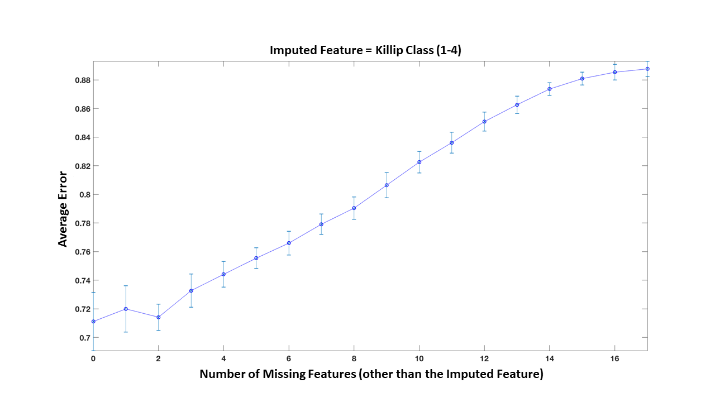


**F**

Binary Variables:


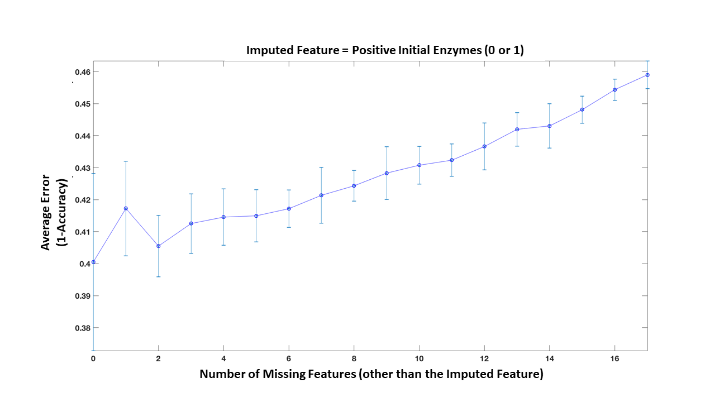


**K**


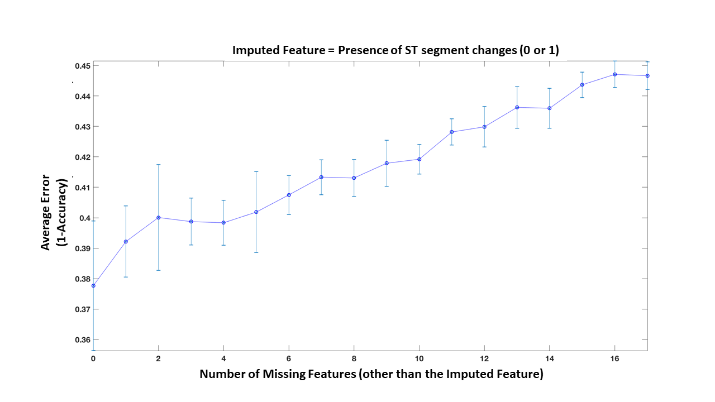


**H**


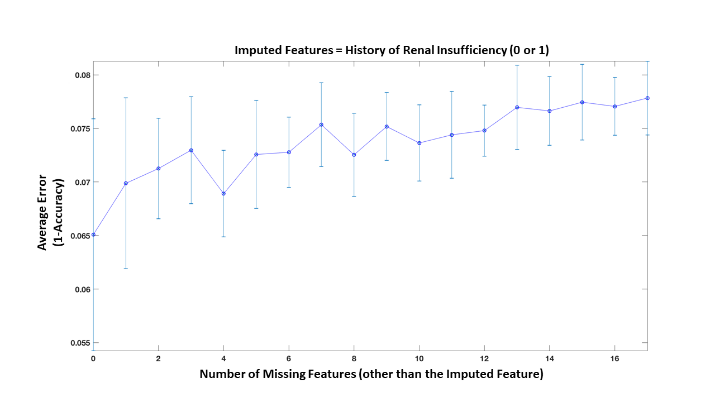


**J**


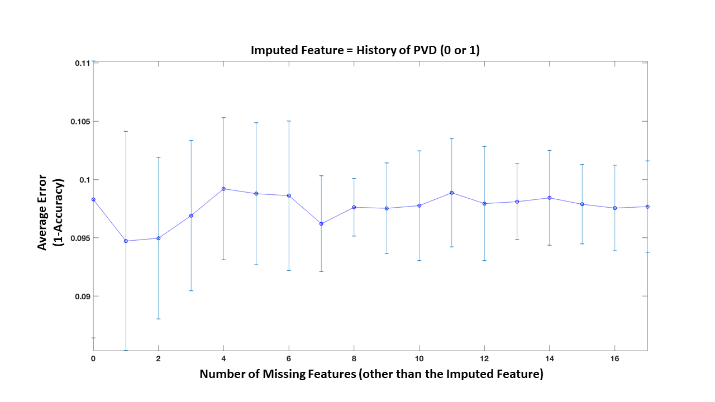


**I**


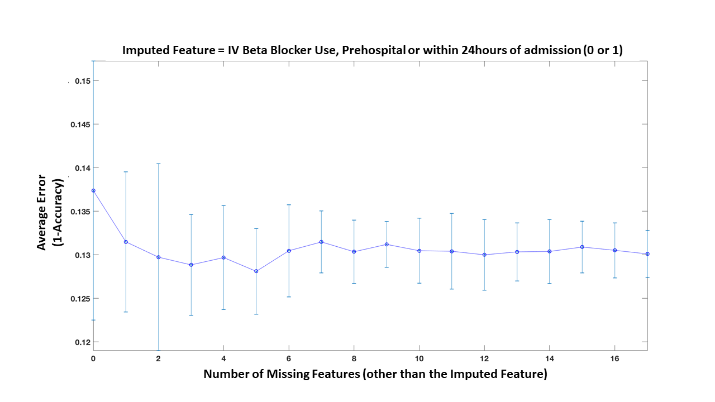


**L**


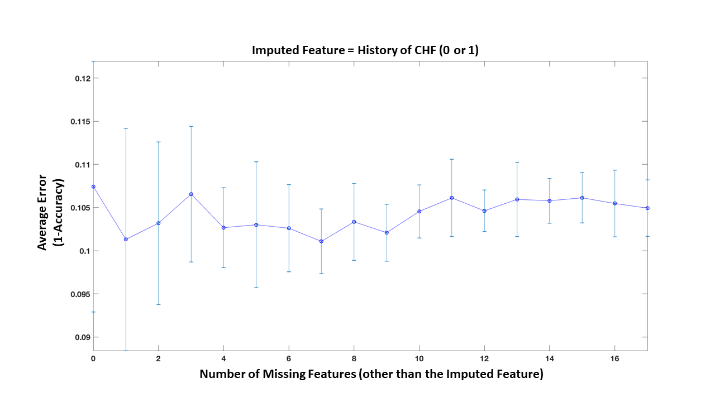


**M**


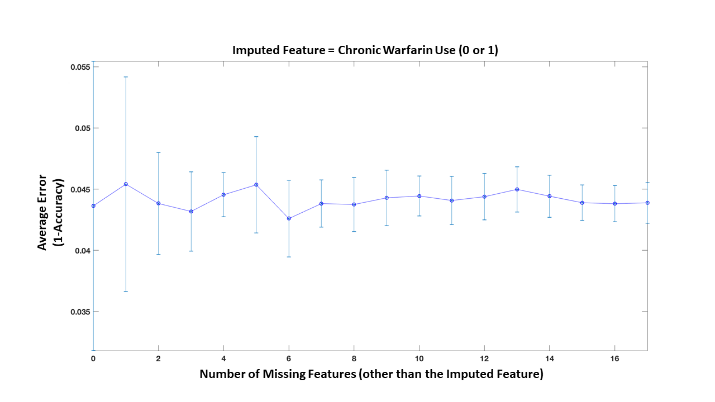


**N**


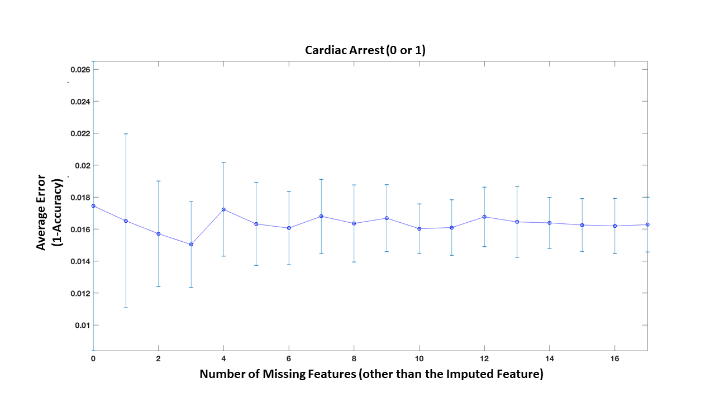


**G**


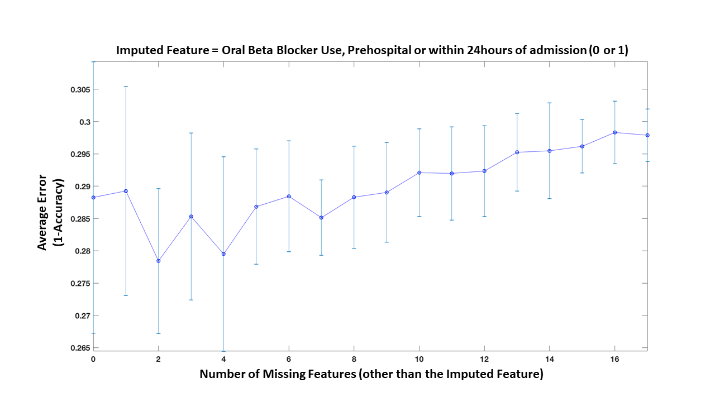


**O**


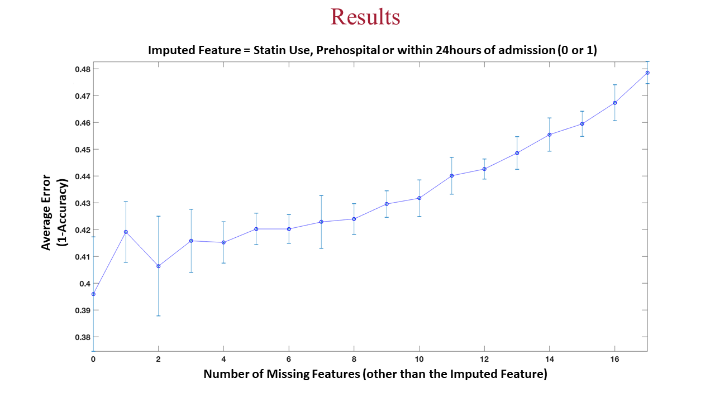


**P**


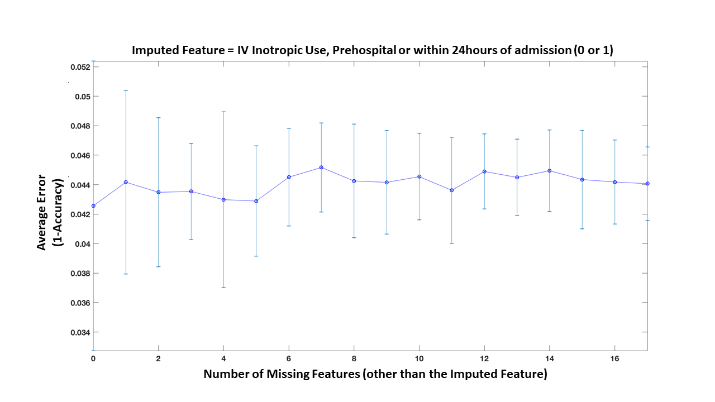


**R**


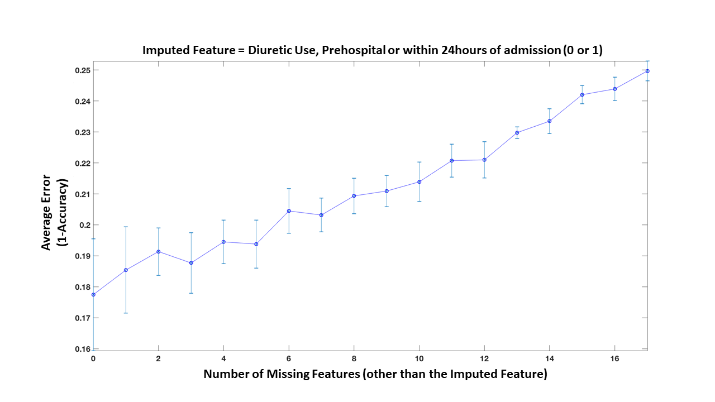


**S**


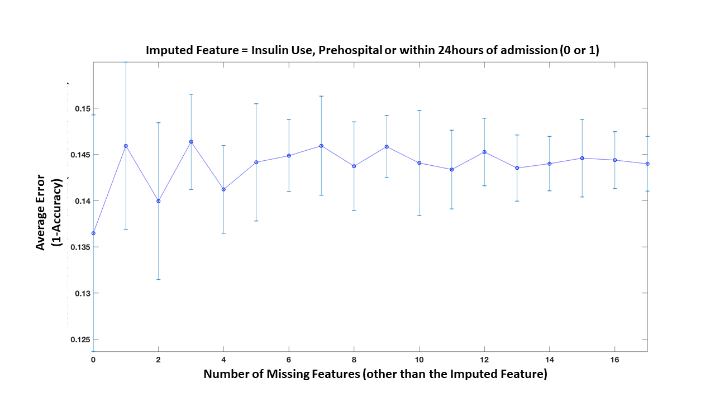


**Q**

**Figure S1**. Error/accuracy for imputing each of the clinical variables in the RLRVI model. Error bars denote the standard deviation.

*Model Parameters*

**Table S1:** Model parameters for each feature and intercept. The weights shown are for normalized features, where the features are normalized using $\frac{X-M}{R}$, where X is the feature and M and R are given in the table. The mean of each feature for the multivariate normal distribution is also shown for the normalized features.

| **#** | **Feature** | **Weight (for Normalized Features)** | **Normalization Factors** | **Mean (Normalized)** |
| --- | --- | --- | --- | --- |
| 0 | Intercept | -0.5752 | N/A | N/A |
| 1 | Age | 3.144 | M: 18.598  R: 86.888 | 0.5407 |
| 2 | Pulse | 1.357 | M: 0  R: 265 | 0.3007 |
| 3 | Systolic Blood Pressure | -1.687 | M: 0  R: 280 | 0.5071 |
| 4 | Initial Creatinine | 2.518 | M: 0  R: 14.700 | 0.08134 |
| 5 | Killip Class | 0.7635 | M: 1  R: 3 | 0.07417 |
| 6 | Cardiac Arrest | -0.5941 | N/A | 0.9834 |
| 7 | Initial Positive Enzymes | -0.3360 | N/A | 0.5320 |
| 8 | ST Segment Deviation | -0.3571 | N/A | 0.4518 |
| 9 | Admission Weight (kg) | -1.572 | M: 22  R: 205 | 0.2767 |
| 10 | History of Renal Insufficiency | -0.2052 | N/A | 0.9220 |
| 11 | History of Congestive Heart Failure | -0.4302 | N/A | 0.8949 |
| 12 | History of Peripheral Vascular Disease | -0.2197 | N/A | 0.9029 |
| 13 | Warfarin - Chronic | -0.2595 | N/A | 0.9550 |
| 14 | Oral Beta Blocker - Pre-Hospital or within 1^st^ 24 Hours | 0.2851 | N/A | 0.3019 |
| 15 | Statin - Pre-Hospital or within 1^st^ 24 Hours | 0.3568 | N/A | 0.4897 |
| 16 | Diuretic - Pre-Hospital or within 1^st^ 24 Hours | -0.4436 | N/A | 0.7467 |
| 17 | Insulin - Pre-Hospital or within 1^st^ 24 Hours | -0.2925 | N/A | 0.8572 |
| 18 | IV Inotropic Agent - Pre-Hospital or within 1^st^ 24 Hours | -1.245 | N/A | 0.9545 |
| 19 | IV Beta Blocker - Pre-Hospital or within 1^st^ 24 Hours | -0.1558 | N/A | 0.8706 |

**Table S2: Covariance matrix for the multivariate normal distribution for the normalized features (divided into three tables). Feature numbers correspond to those in Table S1.**

|  | **1** | **2** | **3** | **4** | **5** | **6** |
| --- | --- | --- | --- | --- | --- | --- |
| **1** | 2.309E-02 | 8.294E-04 | 8.639E-04 | 9.622E-04 | 5.668E-03 | 2.596E-04 |
| **2** | 8.294E-04 | 6.407E-03 | 9.582E-04 | 2.772E-04 | 3.273E-03 | -4.074E-04 |
| **3** | 8.639E-04 | 9.582E-04 | 1.120E-02 | -1.406E-04 | -8.880E-04 | 1.042E-03 |
| **4** | 9.622E-04 | 2.772E-04 | -1.406E-04 | 3.231E-03 | 1.326E-03 | -1.367E-04 |
| **5** | 5.668E-03 | 3.273E-03 | -8.880E-04 | 1.326E-03 | 3.343E-02 | -2.639E-03 |
| **6** | 2.596E-04 | -4.074E-04 | 1.042E-03 | -1.367E-04 | -2.639E-03 | 1.633E-02 |
| **7** | -5.187E-03 | -4.677E-03 | 2.631E-03 | -1.328E-03 | -7.466E-03 | 1.146E-03 |
| **8** | 1.485E-03 | -2.805E-03 | 2.601E-03 | 1.733E-04 | -5.501E-03 | 3.391E-03 |
| **9** | -3.905E-03 | -9.088E-05 | 5.743E-04 | -5.655E-05 | -1.210E-03 | -4.894E-06 |
| **10** | -4.952E-03 | -1.143E-03 | -1.101E-04 | -7.015E-03 | -5.537E-03 | -2.737E-04 |
| **11** | -9.715E-03 | -2.907E-03 | 7.053E-04 | -2.704E-03 | -1.513E-02 | -1.656E-04 |
| **12** | -5.423E-03 | -9.573E-04 | -4.352E-04 | -1.882E-03 | -4.342E-03 | -4.041E-04 |
| **13** | -3.381E-03 | -8.400E-04 | 4.823E-04 | -5.421E-04 | -2.258E-03 | -4.127E-06 |
| **14** | 7.115E-03 | 1.460E-03 | -4.184E-03 | 1.151E-03 | 1.383E-02 | -2.837E-03 |
| **15** | 6.344E-03 | 2.575E-03 | -2.726E-04 | 9.113E-04 | 7.136E-03 | -1.065E-03 |
| **16** | -1.758E-02 | -6.697E-03 | -8.186E-04 | -3.352E-03 | -3.126E-02 | 1.205E-03 |
| **17** | -2.407E-03 | -3.158E-03 | -1.054E-03 | -2.037E-03 | -8.528E-03 | 1.414E-03 |
| **18** | -1.430E-03 | -8.982E-04 | 3.870E-03 | -8.829E-04 | -9.005E-03 | 4.563E-03 |
| **19** | 3.769E-03 | -3.337E-03 | -2.748E-03 | 2.259E-05 | 2.208E-03 | 8.000E-04 |

|  | **7** | **8** | **9** | **10** | **11** | **12** |
| --- | --- | --- | --- | --- | --- | --- |
| **1** | -5.187E-03 | 1.485E-03 | -3.905E-03 | -4.952E-03 | -9.715E-03 | -5.423E-03 |
| **2** | -4.677E-03 | -2.805E-03 | -9.088E-05 | -1.143E-03 | -2.907E-03 | -9.573E-04 |
| **3** | 2.631E-03 | 2.601E-03 | 5.743E-04 | -1.101E-04 | 7.053E-04 | -4.352E-04 |
| **4** | -1.328E-03 | 1.733E-04 | -5.655E-05 | -7.015E-03 | -2.704E-03 | -1.882E-03 |
| **5** | -7.466E-03 | -5.501E-03 | -1.210E-03 | -5.537E-03 | -1.513E-02 | -4.342E-03 |
| **6** | 1.146E-03 | 3.391E-03 | -4.894E-06 | -2.737E-04 | -1.656E-04 | -4.041E-04 |
| **7** | 2.490E-01 | 3.466E-02 | 1.467E-03 | 4.957E-03 | -1.336E-04 | -6.105E-04 |
| **8** | 3.466E-02 | 2.477E-01 | 2.921E-03 | -5.645E-03 | -1.117E-02 | -3.336E-03 |
| **9** | 1.467E-03 | 2.921E-03 | 7.673E-03 | 6.339E-04 | 8.358E-04 | 1.096E-03 |
| **10** | 4.957E-03 | -5.645E-03 | 6.339E-04 | 7.194E-02 | 1.762E-02 | 1.172E-02 |
| **11** | -1.336E-04 | -1.117E-02 | 8.358E-04 | 1.762E-02 | 9.404E-02 | 1.100E-02 |
| **12** | -6.105E-04 | -3.336E-03 | 1.096E-03 | 1.172E-02 | 1.100E-02 | 8.765E-02 |
| **13** | -1.974E-03 | -4.656E-03 | -1.605E-04 | 3.502E-03 | 1.088E-02 | 3.713E-03 |
| **14** | -1.622E-03 | -1.052E-02 | -2.925E-03 | -2.013E-03 | -7.500E-03 | -5.599E-03 |
| **15** | 1.439E-03 | -7.505E-03 | -3.252E-03 | -5.499E-04 | -4.742E-03 | 4.843E-04 |
| **16** | 1.291E-02 | -4.998E-03 | 6.908E-04 | 1.948E-02 | 4.802E-02 | 1.494E-02 |
| **17** | 8.898E-03 | 2.133E-03 | -2.799E-03 | 1.111E-02 | 1.342E-02 | 9.199E-03 |
| **18** | 7.051E-03 | 1.188E-02 | 8.477E-04 | 1.561E-03 | 2.235E-03 | 7.650E-04 |

|  | **13** | **14** | **15** | **16** | **17** | **18** | **19** |
| --- | --- | --- | --- | --- | --- | --- | --- |
| **1** | -3.381E-03 | 7.115E-03 | 6.344E-03 | -1.758E-02 | -2.407E-03 | -1.430E-03 | 3.769E-03 |
| **2** | -8.400E-04 | 1.460E-03 | 2.575E-03 | -6.697E-03 | -3.158E-03 | -8.982E-04 | -3.337E-03 |
| **3** | 4.823E-04 | -4.184E-03 | -2.726E-04 | -8.186E-04 | -1.054E-03 | 3.870E-03 | -2.748E-03 |
| **4** | -5.421E-04 | 1.151E-03 | 9.113E-04 | -3.352E-03 | -2.037E-03 | -8.829E-04 | 2.259E-05 |
| **5** | -2.258E-03 | 1.383E-02 | 7.136E-03 | -3.126E-02 | -8.528E-03 | -9.005E-03 | 2.208E-03 |
| **6** | -4.127E-06 | -2.837E-03 | -1.065E-03 | 1.205E-03 | 1.414E-03 | 4.563E-03 | 8.000E-04 |
| **7** | -1.974E-03 | -1.622E-03 | 1.439E-03 | 1.291E-02 | 8.898E-03 | 7.051E-03 | 9.319E-03 |
| **8** | -4.656E-03 | -1.052E-02 | -7.505E-03 | -4.998E-03 | 2.133E-03 | 1.188E-02 | 1.865E-02 |
| **9** | -1.605E-04 | -2.925E-03 | -3.252E-03 | 6.908E-04 | -2.799E-03 | 8.477E-04 | -1.751E-03 |
| **10** | 3.502E-03 | -2.013E-03 | -5.499E-04 | 1.948E-02 | 1.111E-02 | 1.561E-03 | -5.785E-04 |
| **11** | 1.088E-02 | -7.500E-03 | -4.742E-03 | 4.802E-02 | 1.342E-02 | 2.235E-03 | -2.083E-03 |
| **12** | 3.713E-03 | -5.599E-03 | 4.843E-04 | 1.494E-02 | 9.199E-03 | 7.650E-04 | -1.882E-03 |
| **13** | 4.298E-02 | -1.276E-03 | -1.859E-04 | 1.117E-02 | 2.073E-03 | 2.759E-04 | -3.913E-04 |
| **14** | -1.276E-03 | 2.107E-01 | 4.241E-02 | -1.547E-02 | -1.619E-03 | -1.546E-02 | 4.382E-03 |
| **15** | -1.859E-04 | 4.241E-02 | 2.499E-01 | -4.194E-03 | 8.152E-03 | -7.668E-03 | 8.468E-04 |
| **16** | 1.117E-02 | -1.547E-02 | -4.194E-03 | 1.891E-01 | 2.546E-02 | 1.091E-02 | -2.534E-04 |
| **17** | 2.073E-03 | -1.619E-03 | 8.152E-03 | 2.546E-02 | 1.224E-01 | 6.024E-03 | 2.951E-03 |
| **18** | 2.759E-04 | -1.546E-02 | -7.668E-03 | 1.091E-02 | 6.024E-03 | 4.340E-02 | 1.801E-03 |
